# Potential gains from radical treatment of men with prostate cancer according to life expectancy

**DOI:** 10.1002/bco2.70076

**Published:** 2025-09-10

**Authors:** Sandra Irenaeus, Hans Garmo, Rolf Gedeborg, Mats Ahlberg, David Robinson, Pär Stattin, Kerri Beckmann

**Affiliations:** ^1^ Department of Immunology, Genetics and Pathology Uppsala University Uppsala Sweden; ^2^ Regional Cancer Center Midsweden Uppsala Sweden; ^3^ Department of Surgical Sciences Uppsala University Uppsala Sweden; ^4^ Department of Urology, Ryhov Hospital Jonkoping Sweden; ^5^ Cancer Epidemiology and Population Health Research group University of South Australia Adelaide Australia; ^6^ TOUR (Transforming cancer OUtcomes through Research), Kings College London London UK

**Keywords:** age, comorbidity, life expectancy, prostate cancer, radical treatment

## Abstract

**Objectives:**

To investigate the impact of age and life expectancy on treatment decisions and its consequences for outcomes among men with intermediate and high‐risk prostate cancer (PCa).

**Materials and methods:**

We studied men in Prostate Cancer data Base Sweden (PCBaSe) diagnosed between 2008 and 2022 with intermediate‐risk or high‐risk localized or locally advanced PCa and life expectancy between 2.5 and 15 years in the absence of PCa. Estimates of life expectancy were based on age and two comorbidity indices.

**Results:**

A total of 32 196 men were included in the analyses. Of these, 17 419 (54%) had a life expectancy between 10 and 15 years, of whom 11 147 (64%) received primary radical treatment. Age had a stronger influence than life expectancy on the selection of treatment. Around 10% of deaths within 10 years of diagnosis could potentially have been avoided if men with >10 years life expectancy, regardless of age, had received radical treatment, based on assumptions of high treatment efficacy (30% reduction in all‐cause mortality) and high uptake of treatment (90%).

**Conclusion:**

A substantial proportion of healthy older men with intermediate and high‐risk PCa did not undergo radical treatment. According to our model and assumptions, 10% of deaths within 10 years of diagnosis in these men could potentially have been avoided if they had received radical treatment.

## 1 INTRODUCTION

The risk of progression and prostate cancer (PCa) death depends on cancer characteristics, treatment and life expectancy. Risk category and life expectancy are crucial for selection of treatment, and hence the European Association of Urology (EAU) Guidelines, the National Comprehensive Cancer Network (NCCN) guidelines, as well as the Swedish prostate cancer guidelines, recommend that life expectancy be central in clinical decisions on treatment for PCa.^1^, ^2^, ^3^

EAU guidelines recommend radical treatment for men with high‐risk PCa and life expectancy >10 years,^1^ whereas NCCN guidelines recommend radical treatment for men with high‐risk and very high‐risk PCa and life expectancy >5 years.^2^ The Swedish guidelines recommend radical treatment for men with unfavourable intermediate‐risk and high‐risk PCa who have a life expectancy >10 years, and that radical treatment for men with very high‐risk PCa and life expectancy > 5 years should be discussed at a multidisciplinary conference.^3^

The aims of this study were to investigate the impact of age and life expectancy on treatment decisions among men with intermediate and high‐risk localized and locally advanced PCa and its consequences for outcomes.

## 2 MATERIALS AND METHODS

### 2.1 Data sources

The National Prostate Cancer Register (NPCR) of Sweden collects data on tumour characteristics, mode of detection and treatment, enabling studies on treatment and adherence to national guidelines.^4^, ^5^ In Prostate Cancer data Base Sweden (PCBaSe), NPCR has been linked to several health care registers^6^ by use of the unique Swedish Personal Identity Number. In this study, we use data from NPCR, the National Patient Register, the National Prescribed Drug Register and the Cause of Death Register. Reporting of new cases of PCa is mandatory by law to the Swedish Cancer Registry, and PCBaSe is estimated to include 99% of all men registered with PCa between 2008 and 2022 in the Swedish Cancer Register.^7^

### 2.2 Study population

We selected men in PCBaSe diagnosed between January 1, 2008, and June 30, 2021, with unfavourable intermediate‐risk, high‐risk and very high‐risk localized or locally advanced PCa who had a life expectancy between 2.5 and 15 years in the absence of PCa. Men with a life expectancy >15 years were not included in the study, as a large majority of these men should unequivocally receive radical treatment. Men with a life expectancy between 2.5 and 5 years at diagnosis were included to improve the statistical fitting of cubic splines. To allow for treatment decisions to have been made, follow‐up commenced at 180 days after the date of diagnosis and continued until death, emigration, or December 31, 2021, whichever event came first. Risk categories were defined according to a modification of the National Comprehensive Cancer Network Clinical Practice Guidelines in Oncology (NCCN Guidelines) adopted by NPCR that closely resemble the risk stratification in the Swedish National Guidelines for Prostate Cancer:

Unfavourable intermediate‐risk PCa: Gleason score 4 + 3 with PSA < 20 ng/ml or Gleason 6 with PSA > 15–20 ng/ml and stage T1–2.

High‐risk PCa: Gleason 8–10, or 20 ≤ PSA < 50 ng/ml, or stage T3.

Very high‐risk PCa: Any Gleason, stage T4, N1 or 50 ≤ PSA < 100 ng/ml, or 50 ≤ PSA < 500 if M0 according to bone scan.

### 2.3 Exposure

Life expectancy in the absence of PCa was calculated based on age, a Drug Comorbidity Index (DCI)^8^, ^9^ and a Multidimensional Diagnosis‐based Comorbidity Index (MDCI)^10^ according to a previously used method.^11^ The DCI was based on all drug prescriptions registered in the National Prescribed Drug Register within 365 days preceding the date of PCa diagnosis.^8^ The MDCI used all ICD‐10 codes related to hospital discharges registered up to 10 years prior to the date of PCa diagnosis in the Inpatient and Outpatient Registers.^10^ Both comorbidity indices have previously been shown to outperform the Charlson Comorbidity Index (CCI) in predicting death.^8^, ^12^ The estimated life expectancy was calculated as the area under the simulated survival curve.

### 2.4 Outcomes

Radical treatment was defined as radical prostatectomy or radical radiotherapy registered as primary treatment in NPCR.^13^ Primary treatment registered in NPCR has previously been found to correspond closely to the actual primary treatment received.^14^ Men who did not undergo radical treatment were defined as conservatively treated. The outcome in the survival analysis was death from any cause.

### 2.5 Statistical analyses

The probability of radical treatment for PCa was determined for combinations of age and life expectancy. The interaction effect between age at diagnosis and life expectancy on the probability to receive radical treatment was modelled using logistic regression as a restricted two‐dimensional cubic spline via the gam‐function in the R‐package mgcv. These analyses were stratified by risk category and presented in ‘heat maps’ showing probability for radical treatment.

To assess potential gain in survival from radical treatment, we analysed five groups of men where guidelines recommend radical treatment: i) unfavourable intermediate‐risk PCa and life expectancy of 10–15 years; ii) high‐risk PCa and life expectancy of 10–15years; iii) very‐high risk PCa and life expectancy of 10–15 years; iv) high‐risk PCa and life expectancy of 5–10 years; and v) very high‐risk PCa and life expectancy of 5–10 years. Each group was further divided into men who received radical treatment and those who received conservative treatment. In each of these groups, relative survival for men with PCa was compared to PCa‐free men with similar age and comorbidities. This was done by firstly calculating predicted survival curves by simulating survival for PCa‐free men with similar age, DCI and MDCI as men with PCa.^15^ Next, the predicted survival curve was fitted to the observed survival for men with PCa by applying a proportional hazards model. The relative survival for men in each risk category/life expectancy group was determined from the survival curve deviating the least from observed survival at 8–11 years of follow‐up.

The potential life years gained from radical treatment for conservatively treated men with PCa was estimated by calculating the area under the relative survival curves assuming hypothetical reductions in all‐cause mortality of 10%, 20% and 30% with treatment (Figure S1). Confidence intervals were determined by bootstrapping within each group.

We then estimated the proportion of deaths avoided within 10 years for combinations of hypothetical relative reductions in all‐cause mortality (10%, 20% and 30%) with radical treatment and different uptake of radical treatment (100%, 90%, 75% and 60%), by calculating the difference in proportions of deaths from any cause within 10 years between the relative survival curve and the potential gain survival curves.

## 3 RESULTS

### 3.1 Patient characteristics

A total of 32 196 men diagnosed with PCa between 2008 and 2021 were included in the analyses (Figure S2 Flow chart). The median age at diagnosis was 76 years (IQR 73–81) (Table 1). 32% of men in the study cohort had unfavourable intermediate‐risk PCa, 51% high‐risk PCa and 17% very high‐risk PCa. Eight percent of men had a life expectancy between 2.5 and 5 years, 38% had a life expectancy between 5 and 10 years and 54% had a life expectancy between 10 and 15 years and the proportions of men who received radical treatment were 4%, 17% and 64% for men within the life expectancy categories, respectively.

**TABLE 1:** Baseline characteristics of men with unfavourable intermediate risk, high‐risk, and very‐high risk prostate cancer in Prostate Cancer data Base Sweden diagnosed 2008–2022 stratified by life expectancy.

Life expectancy								
	2.5–5 years (n = 2442)		5–10 years (n = 12 335)		10–15 years (n = 17 419)		Any (n = 32 196)	
**Age, median (Q**_**1**_ **‐Q**_**3**_ **)**	84	(80–88)	81	(77–83)	74	(72–76)	76	(73–81)
≤75**, n (%)**	224	(9)	1616	(13)	10 229	(59)	12 069	(37)
76–80	372	(15)	3246	(26)	6856	(39)	10 474	(33)
81–85	635	(26)	5484	(44)	334	(2)	6453	(20)
86+	1211	(50)	1989	(16)			3200	(10)
**MDCI, n (%)**								
**median (Q**_**1**_ **‐Q**_**3**_ **)**	1.2	(0.7–1.8)	0.3	(0.0–0.8)	0.0	(−0.1–0.3)	0.1	(0.0–0.6)
<0	63	(3)	2771	(22)	8425	(48)	11 259	(35)
0–2	928	(38)	7644	(62)	8601	(49)	17 173	(53)
2–4	1012	(41)	1784	(14)	392	(2)	3188	(10)
4+	439	(18)	136	(1)	1	(0)	576	(2)
**DCI**								
**median (Q**_**1**_ **‐Q**_**3**_ **)**	3.5	(2.2–4.8)	1.7	(0.8–2.9)	0.8	(0.3–1.7)	1.3	(0.5–2.4)
−0.8–1, **n (%)**	168	(7)	3832	(31)	9781	(56)	13 781	(43)
1.01–2	345	(14)	3224	(26)	4305	(25)	7874	(24)
2.01–3	479	(20)	2429	(20)	1999	(11)	4907	(15)
3.01–4	503	(21)	1478	(12)	839	(5)	2820	(9)
4+	947	(39)	1372	(11)	495	(3)	2814	(9)
**T‐stage, n (%)**								
T1	583	(24)	3365	(27)	6793	(39)	10 741	(33)
T2	931	(38)	5120	(42)	6854	(39)	12 905	(40)
T3	816	(33)	3557	(29)	3560	(20)	7933	(25)
T4	112	(5)	293	(2)	212	(1)	617	(2)
**PSA, n (%)**								
0–9.99	330	(14)	2285	(19)	5156	(30)	7771	(24)
10–19.99	726	(30)	4318	(35)	6777	(39)	11 821	(37)
20–49.99	891	(36)	3876	(31)	3838	(22)	8605	(27)
50+	495	(20)	1856	(15)	1648	(9)	3999	(12)
**Gleason, n (%)**								
Gleason 6	233	(10)	1328	(11)	2440	(14)	4001	(12)
Gleason 7 (3 + 4)	453	(19)	2560	(21)	3738	(21)	6751	(21)
Gleason 7 (4 + 3)	599	(25)	3101	(25)	4931	(28)	8631	(27)
Gleason 8	490	(20)	2388	(19)	3024	(17)	5902	(18)
Gleason 9–10	667	(27)	2958	(24)	3286	(19)	6911	(21)
**Risk category, n (%)**								
Unfavourable intermediate‐risk	446	(18)	3158	(26)	6714	(39)	10 318	(32)
High‐risk	1404	(57)	6879	(56)	8215	(47)	16 498	(51)
Very high‐risk	592	(24)	2298	(19)	2490	(14)	5380	(17)
**Mode of** **detection, n (%)**								
Screening	698	(29)	4658	(38)	8650	(50)	14 006	(44)
LUTS	1086	(44)	5114	(41)	5766	(33)	11 966	(37)
Symptoms	603	(25)	2198	(18)	2428	(14)	5229	(16)
Missing	55	(2)	365	(3)	575	(3)	995	(3)
**Curative intention, n (%)**								
Yes	94	(4)	2058	(17)	11 147	(64)	13 299	(41)
No	35	(1)	135	(1)	130	(1)	300	(1)

### 3.2 Probability of radical treatment

**FIGURE 1:**
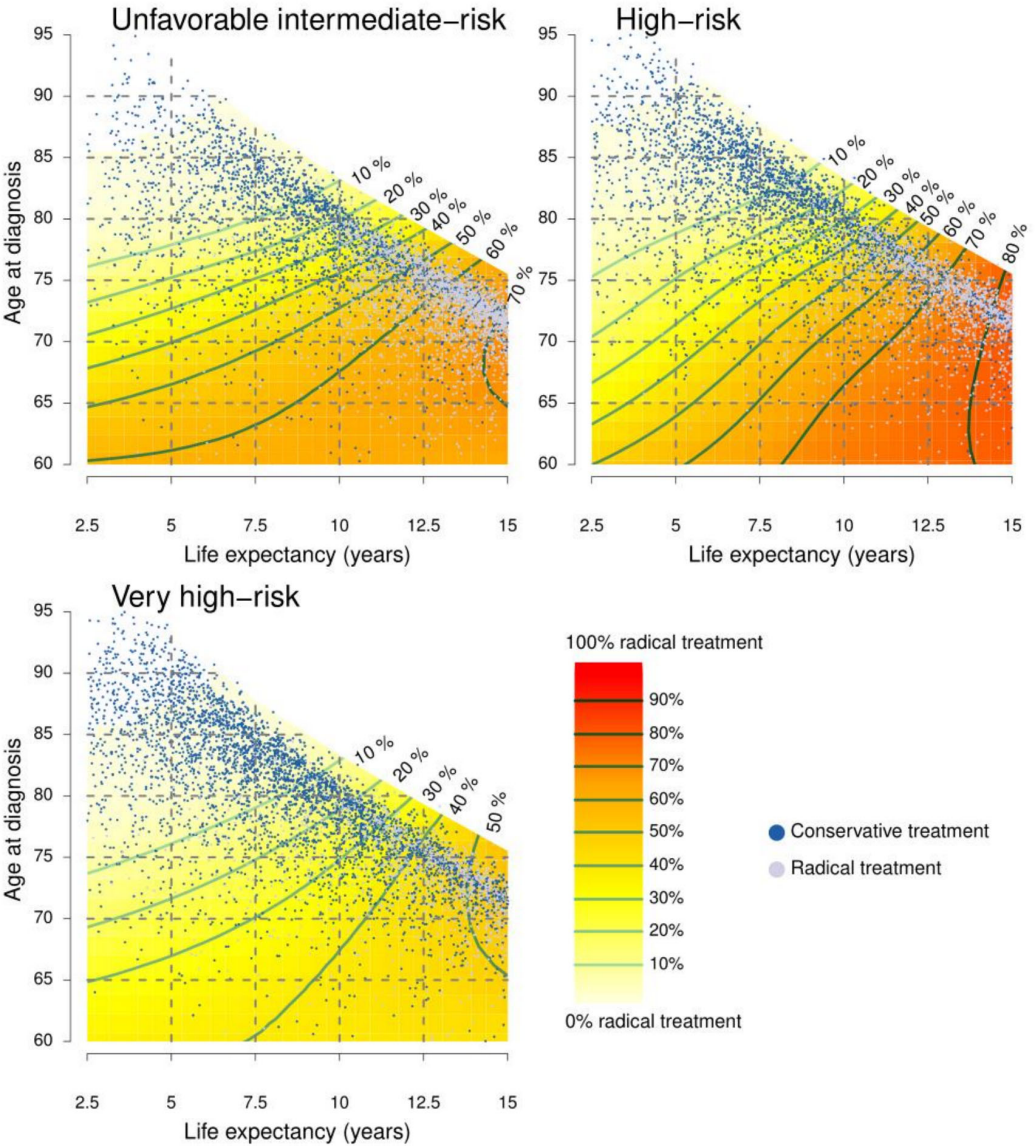
**Probability of radical treatment according to life expectancy and age.** *Combined heat maps and scatterplots depicting probability of radical treatment according to life expectancy and age. Treatment according to age and life expectancy is indicated by dots. Each dot represents one of 5000 randomly selected men in each risk category; grey dots represent radical treatment, and blue dots conservative treatment. Green curves are gradients of probabilities of radical treatment. Light green indicates a low probability for radical treatment. A vertical direction implies that radical treatment was driven by age whereas a horizontal direction implies that treatment was driven by life expectancy.*

For a given life expectancy, the probability of radical treatment decreased with age. For example, for men with unfavourable intermediate‐risk PCa and a life expectancy of 10 years, the probability of radical treatment was <40% for men aged above 75 years, <30% for men aged above 77 years and <20% for men aged above 80 years (Figure 1).

### 3.3 Survival analysis

**FIGURE 2:**
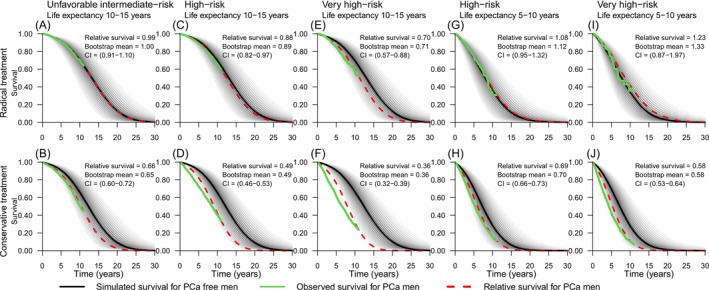
**Observed, expected, and relative survival for men with prostate cancer and matched prostate cancer‐free control men.** *Green lines depict observed survival for men with prostate cancer (PCa). Black lines depict expected survival based on PCa‐free men with the same age, CCI and DCI as the PCa men. Grey lines depict relative survival curves with HR adjustments ranging from 0.5 to 2. Red lines depict the relative survival curve with least deviation from the observed survival curve at 8–11 years of follow‐up.*

Figure 2 shows observed, simulated and relative survival for men with PCa and PCa‐free men with similar life expectancy. For men who underwent radical treatment, the relative survival ranged between 0.71 and 1.33, with the highest relative survival seen for men with very high‐risk PCa and a life expectancy of 10–15 years. The relative survival ranged between 0.36 and 0.70 for conservatively treated men and was lowest for men with very high‐risk PCa with a life expectancy of 10–15 years. Good agreement between observed and relative survival was found in most strata. The largest difference was seen in conservatively treated men with high‐risk and very high‐risk PCa for whom observed survival was substantially shorter than the relative survival.

### 3.4 Potential years of life gained and proportion of deaths potentially avoidable with curative treatment

According to our model, between 9% and 12% of deaths within 10 years could potentially have been avoided if conservatively treated men had received radical treatment, assuming high efficacy of radical treatment (30% reduction in all‐cause mortality) and high uptake of radical treatment (90%) (Table 2).

**TABLE 2:** **Potential years of life gained and proportion of deaths avoided at 10 years post‐diagnosis**.

		Intermediate‐risk PCa life expectancy 10–15 years	High‐risk PCa life expectancy 10–15 years	Very high‐risk PCa life expectancy 10–15 years	High‐risk PCa life expectancy 5–10 years	Very high‐risk PCa life expectancy 5–10 years
**Potential years of life gained** assuming all‐cause mortality reduction of:						
10%		0.49 yrs (0.47–0.51)	0.47 yrs (0.45–0.48)	0.45 yrs (0.42–0.47)	0.40 yrs (0.39–0.41)	0.37 yrs (0.35–0.39)
20%		1.05 yrs (1.01–1.10)	1.01 yrs (0.98–1.04)	0.96 yrs (0.91–1.01)	0.87 yrs (0.84–0.90)	0.82 yrs (0.77–0.86)
30%		1.72 yrs (1.65–1.79)	1.65 yrs (1.59–1.70)	1.57 yrs (1.49–1.65)	1.44 yrs (1.39–1.49)	1.35 yrs (1.28–1.43)
**Proportion of deaths avoided within 10 years** assuming						
Reduction in all‐cause mortality	Uptake of Radical Rx					
10%	100%	3.6% (3.4% ‐3.7%)	3.8% (3.8% ‐3.9%)	3.8% (3.7% ‐3.9%)	3.2% (3.1% ‐3.3%)	2.7% (2.4% ‐2.9%)
	90%	3.2% (3.1% ‐3.3%)	3.4% (3.4% ‐3.5%)	3.4% (3.3% ‐3.5%)	2.8% (2.7% ‐2.9%)	2.4% (2.1% ‐2.6%)
	75%	2.6% (2.6% ‐2.7%)	2.8% (2.8% ‐2.9%)	2.8% (2.8% ‐2.9%)	2.3% (2.2% ‐2.4%)	1.9% (1.7% ‐2.1%)
	60%	2.1% (2.0% ‐2.2%)	2.3% (2.2% ‐2.3%)	2.2% (2.2% ‐2.3%)	1.8% (1.8% ‐1.9%)	1.5% (1.4% ‐1.7%)
20%	100%	7.4% (7.1% ‐7.6%)	8.0% (7.9% ‐8.1%)	8.1% (8.0% ‐8.2%)	7.0% (6.8% ‐7.2%)	6.0% (5.5% ‐6.5%)
	90%	6.6% (6.4% ‐6.8%)	7.2% (7.1% ‐7.2%)	7.2% (7.1% ‐7.3%)	6.2% (6.0% ‐6.4%)	5.3% (4.8% ‐5.7%)
	75%	5.4% (5.2% ‐5.6%)	5.9% (5.8% ‐5.9%)	5.9% (5.8% ‐6.0%)	5.0% (4.8% ‐5.2%)	4.2% (3.8% ‐4.6%)
	60%	4.3% (4.2% ‐4.4%)	4.6% (4.6% ‐4.7%)	4.6% (4.5% ‐4.7%)	3.9% (3.7% ‐4.0%)	3.3% (3.0% ‐3.6%)
30%	100%	11.5% (11.0% ‐11.9%)	12.6% (12.4% ‐12.8%)	13.0% (12.9% ‐13.1%)	11.6% (11.3% ‐11.9%)	10.2% (9.4% ‐10.9%)
	90%	10.2% (9.8% ‐10.5%)	11.2% (11.0% ‐11.3%)	11.5% (11.4% ‐11.5%)	10.1% (9.8% ‐10.4%)	8.8% (8.1% ‐9.5%)
	75%	8.4% (8.1% ‐8.6%)	9.1% (9.0% ‐9.2%)	9.3% (9.2% ‐9.4%)	8.1% (7.8% ‐8.3%)	7.0% (6.4% ‐7.5%)
	60%	6.6% (6.4% ‐6.8%)	7.2% (7.1% ‐7.2%)	7.2% (7.1% ‐7.3%)	6.2% (6.0% ‐6.4%)	5.3% (4.8% ‐5.7%)

Abbreviations: HR: hazard ratio;

Rx: treatment.

## 4 DISCUSSION

### 4.1 Key results

In this population‐based nationwide cohort study in Sweden of men with intermediate or high‐risk localized or locally advanced PCa diagnosed in 2008–2022 who were potentially eligible for radical treatment, age had a stronger influence than life expectancy on the selection of treatment, despite guidelines recommendations to take larger consideration to life expectancy. According to our models, 10% of deaths in older men with these cancers could potentially have been avoided if more weight had been placed on life expectancy.

### 4.2 Strengths and limitations

Strengths of our study include the population‐based design that includes virtually all men in these PCa risk categories in an entire nation and a very high capture rate of radical treatment.^14^ Furthermore, assessment of life expectancy was based on two newly created highly granulated comorbidity indices: the Drug Comorbidity Index (DCI)^8^, ^9^ and the Multidimensional Diagnosis‐based Comorbidity Index (MDCI)^10^¨ which both outperform the commonly used Charlson Comorbidity Index.^10^

Limitations include estimates of life expectancy did not include data on diagnosis from primary care, but for almost all chronic diseases that affect life expectancy, these diseases are treated with drugs and hence captured by the DCI. Furthermore, we did not have measures of lifestyle factors and frailty, such as grip strength, chair standing or balance testing, which are important predictors of life expectancy in older people.^16^

### 4.3 Interpretation

The high relative survival of radically treated men, sometimes above 1, suggests that men who received radical treatment were highly selected, as treatment efficacy is not 100%. A substantial proportion of men with high‐risk and very high‐risk PCa with estimated life expectancy > 10 years according to our measures did not receive radical treatment despite guideline recommendations.^4^, ^5^ Estimates of life expectancy in old men are notoriously difficult in clinical practice, and these results indicate that clinicians may often underestimate life expectancy.

The observed survival in conservatively treated men with high‐risk/very high‐risk PCa and a life expectancy above 10 years deviated sharply from the relative survival. This could, in part, be explained by undetected metastatic disease in these men. In contrast, most men selected for radical treatment will undergo staging imaging.^17^

### 4.4 Use of radical treatment in older men and ensuing effect on outcome in previous studies

Our model indicates that around 10% of deaths, mostly from PCa, could possibly have been avoided if conservatively treated men who had >10 years life expectancy according to our measures had received radical treatment.

Use of radical treatment among older men in other Nordic countries has been documented to have increased sharply with a concomitant modest decrease in mortality.^18^, ^19^ Of note, Swedish guidelines emphasize that biological age and not chronological age should inform treatment decisions. Thus, adherence to national guidelines will lead to increased use of radical treatment among healthy old men with an ensuing increase in survival.^20^ A 32% risk reduction in all‐cause mortality was found for radical radiotherapy and ADT vs ADT only for locally advanced PCa in The Scandinavian Prostate Cancer Group Study (SPCG) 7^21^ and in SPCG‐4 there was a 14% reduction in all‐cause mortality for radical prostatectomy vs watchful waiting in men ≥65 years of age.^22^

In other studies of old men with PCa, results have been quite similar.^23^, ^24^ For instance, a review of studies on radical radiotherapy among older men with PCa indicated increased survival, especially for those with high‐risk PCa and little comorbidity.^23^ In a previous study in PCBaSe, there was an increase in the use of radical treatment in men with locally advanced PCa from 15% in 2000–2003 to 43% in 2012–2016, and the ensuing decrease in risk of PCa death was most pronounced in men 65–74 years.^24^ The hypothesized relative reductions of 10–30% in all‐cause mortality with radical treatment that we applied in our model are in line with the results in these studies. However, our result of a 9–12 % decrease in all‐cause mortality could be an overestimation as treatment effect seems to decline with older age.

### 4.5 Adverse effects of radical treatment in older men

Radical treatment is often avoided in older men irrespective of life expectancy due to concerns about adverse effects and perceived lack of survival benefit, and it is also likely that some older healthy men themselves actively select conservative treatment.^25^ However, given the recent advancements in prostate cancer treatment, the benefit/risk balance is shifting in favour of radical treatment to treat high‐risk PCa, also in old men. For example, intensity modulated radiotherapy, now commonly used, is associated with fewer gastrointestinal and genitourinary toxicities^26^ than the previously used 3D‐conformal radiotherapy. Moreover, the risk of adverse effects from radiotherapy or decrease in quality of life was not a function of age according to some studies.^27^, ^28^, ^29^

We argue that our results are generalizable to countries and regions with similar health care standards as in Sweden.

## 5 CONCLUSION

Despite guideline recommendations, chronological age had a stronger influence than life expectancy on the selection of radical treatment in old men with intermediate and high‐risk localized or locally advanced PCa in Sweden. A substantial proportion of men with these cancers who were old but had long life expectancy received conservative treatment. According to our model, 10% of deaths in these men could potentially have been avoided if decisions on radical treatment had put more weight on life expectancy than on age.

## AUTHOR CONTRIBUTIONS

*Conception and design*: Sandra Irenaeus, Hans Garmo, Rolf Gedeborg, Pär Stattin, andKerri Beckmann. *Acquisition of data*: Hans Garmo, Rolf Gedeborg, and Pär Stattin. *Analysis and interpretation of data*: Sandra Irenaeus, Hans Garmo, Rolf Gedeborg, PärStattin, and Kerri Beckmann. *Drafting of the manuscript*: Sandra Irenaeus, Pär Stattin, and Kerri Beckmann. *Critical revision of the manuscript for important intellectual content*: Rolf Gedeborg, Mats Ahlberg, and David Robinson. *Statistical analyses*: Hans Garmo, Rolf Gedeborg, and Kerri Beckmann. *Obtaining funding*: Rolf Gedeborg and Pär Stattin. *Administrative, technical, or material support*: Mats Ahlberg, David Robinson, KerriBeckmann, and Pär Stattin. *Supervision*: Pär Stattin and Kerri Beckmann.

## CONFLICT OF INTEREST STATEMENT

The authors report no conflicts of interest.

## DISCLAIMER

Rolf Gedeborg is also employed by the Medical Products Agency (MPA) in Sweden. The MPA is a Swedish Government Agency. The views expressed in this article may not represent the views of the MPA.

## Supporting information


**Figure S1.** **Life years gained assuming different reductions in all‐cause mortality with radical treatment.** Dark shaded lines depict relative survival of PCa men conservatively treated. Lighter shaded lines depict simulated survival assuming reductions in all‐cause mortality with radical treatment of 10%, 20% and 30% respectively. Life years gained was calculated as the difference in area under the survival curves, respectively.

**Figure S2.** Study flow chart.
