# Supplementary figures and images for: Potential gains from radical treatment of men with prostate cancer according to life expectancy
Source: BJUI Compass. 2025 Sep 10;6(9):e70076. doi: 10.1002/bco2.70076 (PMC12422882; doi:10.1002/bco2.70076)

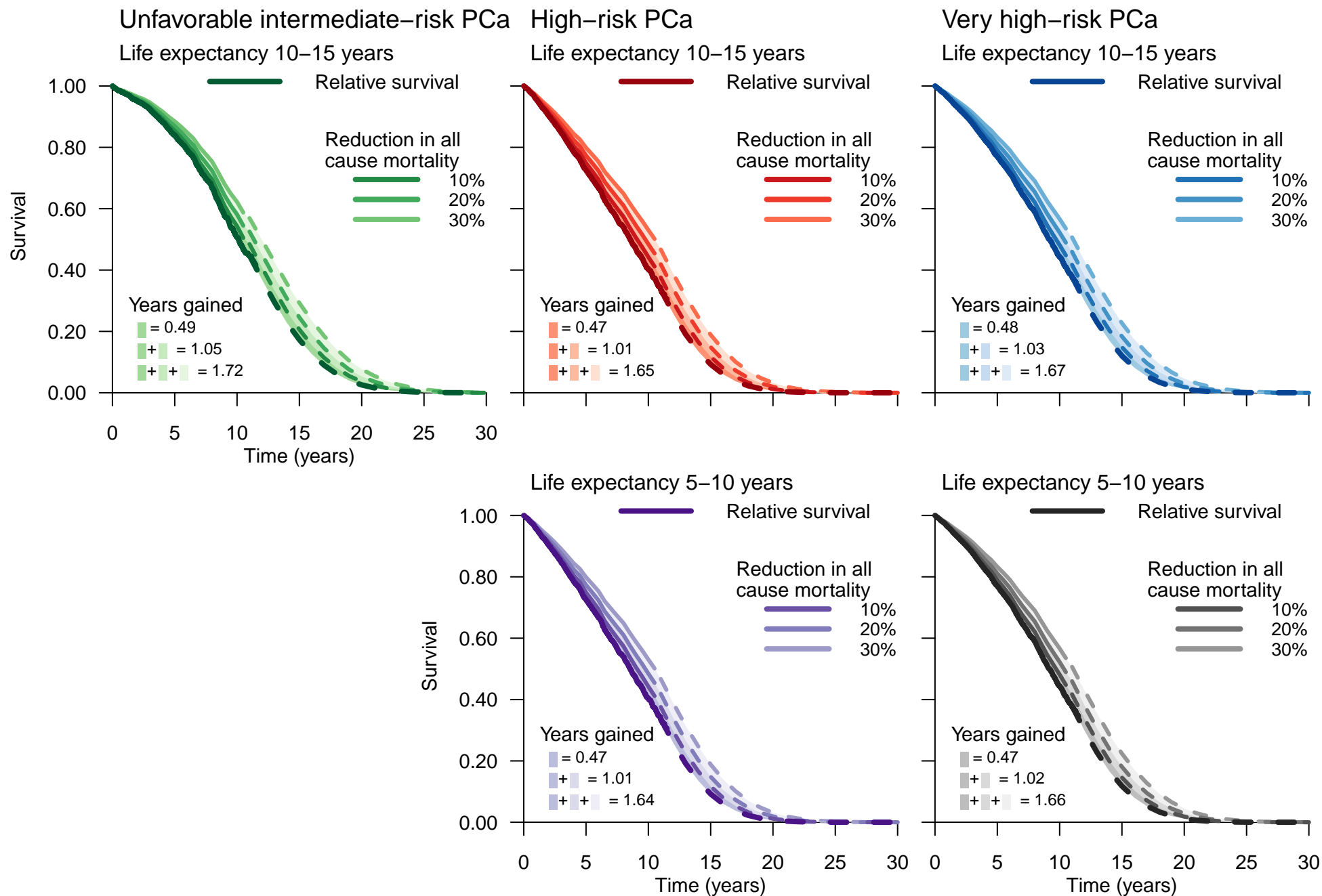

Supplement: Supplementary file 1 — Figure S1. Life years gained assuming different reductions in all‐cause mortality with radical treatment. Dark shaded lines depict relative survival of PCa men conservatively treated. Lighter shaded lines depict simulated survival assuming reductions in all‐cause mortality with radical treatment of 10%, 20% and 30% respectively. Life years gained was calculated as the difference in area under the survival curves, respectively. [file BCO2-6-e70076-s002.pdf]
