# Supplementary material for: Potential gains from radical treatment of men with prostate cancer according to life expectancy
Source: BJUI Compass. 2025 Sep 10;6(9):e70076. doi: 10.1002/bco2.70076 (PMC12422882; doi:10.1002/bco2.70076)
Supplement: Supplementary file 2 — Figure S2. Study flow chart. [file BCO2-6-e70076-s001.docx]

**Life expectancy > 15 years or**

**life expectancy < 2.5 years**

**(n=** **26 268)**

**Follow up shorter than 6 months (n=** **5139)**

**To study**

**(n=** **32 196)**

**Registered in NPCR**

**2008–2022**

**(n=** **152 265)**

**Distant metastases (n=13 707)**

**Low risk prostate cancer (n=37 594)**

**Intermediate risk prostate cancer with either**

- **Gleason score ≤6 and PSA<15 ng/ml**

**or**

- **Gleason score 3+4 and**

**PSA≥10 ng/ml**

**(n=26 475)**

**Missing PSA (n=3615)**

**Missing Gleason score (n=4237)**

**Missing T-stage (n=3034)**
